# Conducting Power of Liquified Gasses

**Published:** 1831

**Authors:** 


2. *Conducting power of Liquefied Gases.*—By making liquefied sulphurous acid gas, a part of the circuit in a galvanic battery of 250 pairs of plates, shocks were received, water was decomposed, and the galvanometer was acted on, as if a continuous metallic communication had existed. Liquid sulphurous acid is therefore an excellent conductor of electricity. Cyanogen, on the contrary, was found to be a perfect non-conductor, even to a voltaic current of from 300 pairs of plates. Liquefied chlorine was also found to be a perfect non-conductor of electricity from a battery of 250 pairs.—*Ibid.*

